# Irregularities of the Teeth and Their Treatment

**Published:** 1886-07

**Authors:** F. E. Howard

**Affiliations:** Buffalo, N. Y.


### IRREGULARITIES OF THE TEETH AND THEIR TREATMENT.

BY F. E. HOWARD, M. D. S., BUFFALO, N. Y.

READ BEFORE A UNION MEETING OF THE SEVENTH AND EIGHTH DISTRICT DENTAL SOCIETIES OF THE STATE OF NEW YORK.

It would be impossible to include all that might be said on this subject in one paper of this kind, or even in a volume. We can only touch upon certain prominent points in the treatment of general cases presented, and the ingenuity of the practitioner must be exercised in carrying out in detail the minor points that have to be observed. No infallible rule can be laid down whereby we can accomplish definite movements of the teeth in a given direction. Judgment, ingenuity, and skill must be exercised. We are thus often taxed to our utmost in accomplishing our desires.

Among the most frequent cases presented are those demanding the bringing down of canines to their proper position, the irregularity being caused by lack of room. This usually requires the extraction of a tooth on either side to accomplish the object, and it must be left to the judgment of the operator which of these shall be selected. It may be the first molar, first or second bicuspid. If one or more of these teeth are extensively decayed, and the others are comparatively sound, it is better to take the weaker ones out, even at the expense of appearance, for it is our duty to accomplish that which is most likely to give the greatest promise of usefulness, as well as comeliness, for the greatest length of time.

Our work as dentists should not be of a temporary character in this field; we must look ahead many years, and weigh carefully in the scales of justice and right that which we believe will be for the greatest benefit to the patients intrusted to our care.

Another very important consideration in this work is to simplify the operation as much as possible. We should not keep young subjects in our hands for months, when by simplifying the operation as many weeks would suffice to accomplish results that are practically as good—where the difference in results would only be noticed by a dentist. Very slight deviation from a normal position does not warrant interference in all cases, particularly in boys' mouths. I have seen cases where the first bicuspid occupied the position of the canine, a strong, well articulated tooth, and the canine pointing out between the first bicuspid and lateral, or the first and second bicuspids. Perhaps, in the majority of cases, we would be warranted in removing the bicuspid and pulling the canine down to place; at other times the extraction of the canine and the cutting off of the inner cusp of the bicuspid and converting it into a canine would be far better. By properly cutting the inner cusp from time to time, a slight elongation will take place that will give it a very natural appearance.

There are also cases where the canine occupies nearly the place of the lateral incisor, and the lateral assumes a very ugly position, being very prominent or depressed, and an attempted change in the position might be doubtful of success. A deviation from nature's arrangement is not always so deplorable as one might imagine. The shaping of the teeth will often cause them to lose their identity in the original type. This can be accomplished by very simple methods, when other changes might involve long, tedious, and complicated operations, with but very slight chances for permanent success. If parents are willing to incur the expense of complicated movements, and the patient is also desirous of obtaining the most perfect results in all the little details, almost anything can be done in this direction.

When we have a narrow arch and desire to expand one or both jaws, I have found the Coffin spring plate the best for general use. The adjustment of a plate of this kind to the upper jaw will also expand the lower by the force of occlusion. The action may be confined to one or both jaws at the will of the dentist, in the construction and adjustment of the plate.

A modification of the Coffin spring will be useful in many different ways; for instance, if the arch is to be expanded in the main, a spring bent in the general form of a W is arranged in position about midway of the plate, and when vulcanized the plate is sawed through the centre, the spring slightly opened and the plate placed in position. From time to time this is spread as the case requires.

When the anterior portion of the arch alone is to be spread, a hinge made of two eyes and a bar, joined and vulcanized into the posterior portion of the plate will hold the posterior part, while the spring will act upon the anterior part alone, or this may be reversed and the back teeth spread at the will of the operator, as the case may require.

Instead of a hinge a more simple method will sometimes answer as well. Drill two holes in the back part of the plate, and with a strong silk ligature or platinum wire bind the parts together, and this will hold them from spreading at this point.

A piano-wire spring, vulcanized into the plate, is also very effectual for moving a tooth out when it is inclined in the mouth and it is not desirable to use a jackscrew. For the movement of canines and bicuspids, a band of platinum with a projecting top or knob cemented to the tooth, is admirable to retain the rubber or silk ligature in position, and to draw the tooth in its proper course, the ligature being attached to some point on the plate, or to a hook attached to some tooth not likely to change position by the force exerted.

For the rotary movement of a tooth, a band of platinum with an arm attached and cemented to the tooth is a powerful agent for twisting such a tooth into position. Also a good and simple method for this movement, as applied to the four anterior teeth, is accomplished by tying a waxed silk thread to any of them, taking two or three turns around the tooth and attaching to a rubber ligature fastened at some convenient point in the plate. The force exerted is in a direction to unwind the ligature from the tooth, and thus it is turned in its socket.

When any one of the six anterior teeth is crowded into the arch, and the space is partly closed so as to prevent the passage of such a tooth out to position, an excellent method is to construct a Coffin spring plate for the expansion of the arch, and while the jaw is being expanded the tooth is forced out with a jackscrew, when the teeth are allowed to return to their original position.

A common class of irregularities of teeth is when one or more of the upper teeth shut upon the lingual surface of the under ones. These I find ordinarily best controlled by making a plate that embraces both jaw and teeth, keeping the teeth partly separated. A band of vulcanite is allowed to extend along the labial surface of the teeth, reaching well up. In this band, opposite the depressed teeth, slots are cut for the retention of elastic bands, and when in position they are looped over the teeth, and the force exerted is in a direction to carry them out to their proper place.

A gold or platinum band vulcanized into the plate, extending around in front of the teeth, will accomplish the same object. When once they have passed over sufficiently far, the plate is to be cut away from the grinding surface of the teeth from time to time, then the occluding force much assists in the work.

In making radical changes in the position of teeth, more or less inflammation will ensue, and often portions of the gum will protrude between the plate and teeth. This must be reduced, from time to time, as the plate or appliance is removed. Aconite and iodine (equal parts) will relieve the inflammation, and a chromic acid solution will dispose of the hypertrophy. If it be quite prominent, excise this portion and apply the chromic acid, which will cause it to quickly assume a normal aspect.

Protrusion of the lawer jaw can ordinarily by corrected in young subjects easily by making a chin cap or pan of brass, swaged to fit the chin, with two eye loops about two inches apart on either side. These are attached to a cap worn upon the head by strong rubber bands running above and below the ear. The upper bands mainly hold the apparatus in position, while the lower ones principally do the work of carrying the jaw back.

When there is excessive development in the tooth, it may be advisable to extract one or more on either side, and carry the anterior ones back, by the usual methods. The circumstances in the case will entirely govern the operation.

